# Supplementary material for: Osteopathy Referrals to and from General Practitioners: Secondary Analysis of Practitioner Characteristics from an Australian Practice-Based Research Network
Source: Healthcare (Basel). 2023 Dec 25;12(1):48. doi: 10.3390/healthcare12010048 (PMC10778730; doi:10.3390/healthcare12010048)
Supplement: Supplementary file 1 [file healthcare-12-00048-s001.zip › Supplementary Materials File S1.pdf]

Supplementary Materials File S1. Practice characteristics of Australian osteopaths based on sending referrals to or receiving referrals from general practitioners.

	Send referrals to a general practitioner				Receive referrals from a general practitioner			
	Yes (n=878, 88.5%)	No (n=114, 11.5%)	p-value	OR [95%CI]	Yes (n=886, 89.3%)	No (n=106, 10.7%)	p-value	OR [95%CI]
**Practice location**								
Urban practice	727 (73.3%)	93 (9.4%)	0.74	-	725 (73.3%)	95 (9.6%)	0.04	0.52 [0.27, 0.99]
More than one practice location	305 (30.7%)	42 (4.2%)	0.65	-	313 (31.6%)	34 (3.4%)	0.51	-
**Co-located with health professional (‘yes’)**								
Another osteopath	579 (58.4%)	64 (6.5%)	0.04	1.51 [1.02, 2.45]	581 (58.6%)	62 (6.3%)	0.15	-
General Practitioner	65 (6.6%)	7 (0.7%)	0.62	-	70 (7.1%)	2 (0.2%)	0.01	4.46 [1.08, 18.46]
Specialist Medical Practitioner	28 (2.8%)	3 (0.3%)	0.75	-	29 (2.9%)	2 (0.2%)	0.44	-
Podiatrist	130 (13.1%)	17 (1.7%)	0.97	-	136 (13.7%)	11 (1.1%)	0.17	-
Physiotherapist	123 (12.4%)	21 (2.1%)	0.21	-	134 (13.5%)	10 (1.0%)	0.11	-
Exercise Physiologist	108 (10.9%)	16 (1.6%)	0.60	-	111 (11.2%)	13 (1.3%)	0.94	-
Occupational Therapist	14 (1.4%)	5 (0.5%)	0.04	0.35 [0.12, 1.00]	18 (1.8%)	1 (0.1%)	0.44	-
Psychologist	164 (16.5%)	27 (2.7%)	0.20	-	170 (17.1%)	21 (2.1%)	0.88	-
Massage Therapist	437 (44.1%)	64 (6.5%)	0.20	-	442 (44.6%)	59 (5.9%)	0.26	-
Acupuncturist	158 (15.9%)	30 (3.0%)	0.03	0.61 [0.39, 0.96]	160 (16.1%)	28 (2.8%)	0.04	0.61 [0.39, 0.98]
Naturopath	170 (17.1%)	23 (2.3%)	0.83	-	166 (16.7%)	27 (2.7%)	0.10	-
Dietician	61 (6.1%)	11 (1.1%)	0.30	-	66 (6.7%)	6 (0.6%)	0.50	-
Nutritionist	66 (6.7%)	12 (1.2%)	0.26	-	70 (7.1%)	8 (0.8%)	0.90	-
**Send referrals to a health professional (‘yes’)**								
Another osteopath	470 (47.4%)	36 (3.6%)	<0.01	2.50 [1.64, 3.78]	459 (46.3%)	47 (4.7%)	0.14	-
General Practitioner	-	-	-	-	810 (81.7%)	68 (6.9%)	<0.01	5.95 [3.75, 9.45]
Specialist Medical Practitioner	422 (42.5%)	21 (2.1%)	<0.01	4.10 [2.50, 6.70]	406 (40.9%)	37 (3.7%)	0.03	1.57 [1.03, 2.40]
Podiatrist	613 (61.8%)	38 (3.8%)	<0.01	4.62 [3.05, 7.00]	594 (59.6%)	57 (5.7%)	<0.01	1.74 [1.16, 2.62]
Physiotherapist	311 (31.4%)	20 (2.0%)	<0.01	2.58 [1.56, 4.26]	307 (30.9%)	24 (2.4%)	0.01	1.81 [1.12, 2.91]
Exercise Physiologist	370 (37.3%)	28 (2.8%)	<0.01	2.23 [1.43, 3.50]	371 (37.4%)	27 (2.7%)	<0.01	2.11 [1.33, 3.33]
Occupational Therapist	103 (10.4%)	3 (0.3%)	<0.01	4.91 [1.53, 15.76]	101 (10.2%)	5 (0.5%)	0.03	2.60 [1.03, 6.53]
Psychologist	330 (33.3%)	19 (1.9%)	<0.01	3.01 [1.80, 5.02]	320 (32.3%)	29 (2.9%)	0.07	-
Massage Therapist	613 (62.8%)	58 (5.8%)	<0.01	2.23 [1.50, 3.13]	606 (61.1%)	65 (6.6%)	0.14	-
Acupuncturist	417 (42.0%)	34 (3.4%)	<0.01	2.12 [1.39, 3.24]	409 (41.2%)	42 (4.2%)	0.20	-
Naturopath	446 (45.0%)	31 (3.1%)	<0.01	2.76 [1.79, 4.26]	428 (43.1%)	49 (4.9%)	0.68	-
Dietician	158 (15.9%)	9 (0.9%)	<0.01	2.56 [1.26, 5.16]	157 (15.8%)	10 (1.0%)	0.03	2.07 [1.05, 4.05]
Nutritionist	118 (11.9%)	11 (1.1%)	0.26	-	119 (12.0%)	10 (1.0%)	0.25	-
**Receive referrals from a health professional (‘yes’)**								
Osteopath	565 (57.0%)	49 (4.9%)	<0.01	2.39 [1.61, 3.55]	570 (57.5%)	44 (4.4%)	<0.01	2.54 [1.68, 3.83]
General Practitioner	810 (81.7%)	76 (7.7%)	<0.01	5.95 [3.75, 9.44]	-	-	-	-
Specialist Medical Practitioner	226 (2.8%)	11 (1.1%)	<0.01	3.24 [1.71, 6.15]	231 (23.3%)	6 (0.6%)	<0.01	5.87 [2.54, 13.58]
Podiatrist	438 (44.2%)	33 (3.3%)	<0.01	2.44 [1.59, 3.74]	250 (25.2%)	16 (1.6%)	<0.01	2.11 [1.27, 3.83]

Physiotherapist	243 (24.5%)	23 (2.3%)	0.09	-	243 (24.5%)	15 (1.5%)	<0.01	2.29 [1.30, 4.04]
Exercise Physiologist	242 (24.4%)	16 (1.6%)	<0.01	2.33 [1.34, 4.03]	61 (6.1%)	0	<0.01	0.88 [0.86, 0.91]
Occupational Therapist	56 (5.6%)	5 (0.5%)	0.40	-	46 (4.6%)	15 (1.5%)	<0.01	3.65 [2.01, 6.63]
Psychologist	139 (14.0%)	15 (1.5%)	0.45	-	144 (14.5%)	10 (1.0%)	0.07	-
Massage Therapist	681 (68.6%)	73 (7.4%)	<0.01	1.94 [1.28, 2.94]	695 (70.1%)	59 (5.9%)	<0.01	2.89 [1.91, 4.39]
Acupuncturist	338 (34.1%)	32 (3.2%)	0.03	1.60 [1.04, 2.47]	337 (34.0%)	33 (3.3%)	0.16	-
Naturopath	367 (37.0%)	33 (3.3%)	<0.01	1.76 [1.15, 2.70]	360 (36.3%)	40 (4.0%)	0.56	-
Dietician	36 (3.6%)	3 (0.3%)	0.45	-	38 (3.8%)	1 (0.1%)	0.11	-
Nutritionist	51 (5.1%)	4 (0.4%)	0.31	-	51 (5.1%)	4 (0.4%)	0.40	-
**Diagnostic imaging (‘yes’)**								
Referral for imaging (‘often’)	65 (6.6%)	8 (0.8%)	0.88	-	39 (3.9%)	34 (3.4%)	0.29	-
Investigation of unknown pathologies	665 (67.0%)	77 (7.8%)	0.06	-	370 (37.3%)	372 (37.5%)	0.01	1.47 [1.10, 1.96]
Investigation of suspected diagnosis	744 (75.0%)	91 (9.2%)	0.18	-	402 (40.5%)	433 (43.6%)	0.33	-
Investigation of potential fractures	670 (67.5%)	80 (8.1%)	0.15	-	373 (37.6%)	377 (38.0%)	0.01	1.45 [1.08, 1.95]
Rule out risk factors prior to treatment	249 (25.1%)	23 (2.3%)	0.06	-	136 (13.7%)	136 (13.7%)	0.33	-
General screening of the spine	27 (2.7%)	5 (0.5%)	0.45	-	9 (0.9%)	23 (2.3%)	0.03	0.42 [0.19, 0.92]
**Patient assessment (‘yes’)**								
Orthopaedic testing	863 (87.0%)	105 (10.6%)	<0.01	4.93 [2.10, 11.54]	67 (6.8%)	6 (0.6%)	0.48	-
Clinical assessment algorithm	419 (42.2%)	49 (4.9%)	0.34	-	673 (67.8%)	69 (7.0%)	0.01	1.69 [1.10, 2.60]
Neurological testing	819 (82.6%)	99 (10.0%)	0.01	2.10 [1.15, 3.85]	748 (75.4%)	87 (8.8%)	0.53	-
Screening questionnaire	561 (56.6%)	72 (7.3%)	0.87	-	680 (68.5%)	70 (7.1%)	0.01	1.70 [1.10, 2.61]
Cranial nerve testing	616 (62.1%)	56 (5.6%)	<0.01	2.43 [1.64, 3.61]	245 (24.7%)	27 (2.7%)	0.63	-
**Information management (‘yes’)**								
Share with eHealth system	2 (0.2%)	0	0.61	-	2 (0.2%)	0	0.62	-
Use Medicare EasyClaim	394 (39.7%)	37 (3.7%)	0.01	1.69 [1.12, 2.56]	417 (42.0%)	14 (1.4%)	<0.01	5.84 [3.28, 10.41]
