# Supplementary material for: Osteopathy Referrals to and from General Practitioners: Secondary Analysis of Practitioner Characteristics from an Australian Practice-Based Research Network
Source: Healthcare (Basel). 2023 Dec 25;12(1):48. doi: 10.3390/healthcare12010048 (PMC10778730; doi:10.3390/healthcare12010048)
Supplement: Supplementary file 1 [file healthcare-12-00048-s001.zip › Supplementary Materials File S2.pdf]

Supplementary Materials File S2. Clinical management characteristics of Australian osteopaths based on sending referrals to or receiving referrals from GPs

	**Send referrals to a general practitioner**				**Receive referrals from a general practitioner**			
	**Yes (n=878, 88.5%)**	**No (n=114, 11.5%)**	**p-value**	**OR [95%CI]**	**Yes (n=886, 89.3%)**	**No (n=106, 10.7%)**	**p-value**	**OR [95%CI]**
**Discuss with patients ('often')**								
Diet	336 (33.9%)	39 (3.9%)	0.39	-	330 (33.3%)	45 (4.5%)	0.30	-
Smoking and drug use	161 (16.3%)	18 (1.8%)	0.50	-	158 (16.0%)	21 (2.1%)	0.62	-
Physical activity	787 (79.4%)	99 (10.0%)	0.34	-	791 (79.8%)	95 (9.5%)	0.93	-
Occupation Health & Safety	453 (45.8%)	53 (5.4%)	0.29	-	462 (46.7%)	44 (4.4%)	0.03	1.54 [1.03, 2.32]
Pain counselling	235 (23.7%)	31 (3.1%)	0.93	-	242 (24.4%)	24 (2.4%)	0.30	-
Stress	438 (44.3%)	51 (5.2%)	0.28	-	430 (43.5%)	59 (6.0%)	0.17	-
Nutrition	217 (21.9%)	35 (3.5%)	0.17	-	222 (22.4%)	30 (3.0%)	0.47	-
Medication	349 (35.3%)	42 (4.2%)	0.53	-	360 (36.4%)	31 (3.1%)	0.02	1.66 [1.07, 2.58]
**Patient presentations ('often')**								
Neck pain	857 (86.5%)	114 (11.5%)	0.10	-	865 (87.3%)	106 (10.7%)	0.12	-
Thoracic pain	804 (81.1%)	105 (10.6%)	0.87	-	812 (81.9%)	97 (9.8%)	0.93	-
Low back pain	864 (87.3%)	113 (11.4%)	0.19	-	871 (88.0%)	106 (10.7%)	0.21	-
Hip musculoskeletal pain	665 (67.2%)	79 (8.0%)	0.12	-	675 (68.2%)	69 (7.0%)	0.01	1.73 [1.13, 2.66]
Knee musculoskeletal pain	442 (44.7%)	49 (5.0%)	0.13	-	450 (45.5%)	41 (4.1%)	0.01	1.65 [1.09, 2.50]
Ankle musculoskeletal pain	303 (30.6%)	30 (3.0%)	0.08	-	300 (30.3%)	33 (3.3%)	0.56	-
Foot musculoskeletal pain	266 (26.9%)	28 (2.8%)	0.20	-	265 (26.8%)	29 (2.9%)	0.58	-
Shoulder musculoskeletal pain	715 (72.3%)	86 (8.7%)	0.11	-	723 (73.1%)	78 (7.9%)	0.40	1.62 (1.02, 2.58]
Elbow musculoskeletal pain	227 (23.0%)	24 (2.4%)	0.27	-	231 (23.4%)	20 (2.0%)	0.11	-
Wrist musculoskeletal pain	171 (17.3%)	17 (1.7%)	0.24	-	168 (17.0%)	20 (2.0%)	0.97	-
Hand musculoskeletal pain	109 (11.1%)	12 (1.2%)	0.57	-	108 (11.0%)	13 (1.3%)	0.99	-
Postural disorders	601 (60.8%)	74 (7.5%)	0.42	-	612 (61.9%)	63 (6.4%)	0.04	1.54 [1.02, 2.33]
Degenerative spine conditions	547 (55.3%)	52 (5.3%)	<0.01	2.00 [1.34, 2.94]	560 (56.6%)	39 (3.9%)	<0.01	2.92 [1.92, 4.44]
Headache disorders	785 (79.3%)	107 (10.8%)	0.15	-	798 (80.6%)	94 (9.5%)	0.60	-
Migraine disorders	361 (36.5%)	39 (3.9%)	0.15	-	365 (36.9%)	35 (3.5%	0.10	-
Spine health maintenance	400 (40.5%)	58 (5.9%)	0.30	-	410 (41.5%)	48 (4.9%)	0.81	-
Chronic or persistent pain	562 (56.8%)	68 (6.9%)	0.34	-	575 (58.1%)	55 (5.6%)	<0.01	1.73 [1.15, 2.59]
Tendinopathies	368 (37.2%)	42 (4.2%)	0.29	-	378 (38.2%)	32 (3.2%)	0.01	1.73 [1.12, 2.67]
Temporomandibular joint disorders	162 (16.4%)	21 (2.1%)	0.98	-	164 (16.6%)	19 (1.9%)	0.90	-
Non-musculoskeletal disorders	111 (11.3%)	15 (1.5%)	0.86	-	112 (11.4%)	14 (1.4%)	0.81	-
**Patient subgroups (treat 'often')**								
4 to 18 years of age	252 (25.4%)	18 (1.8%)	<0.01	2.12 [1.26, 3.59]	248 (25.0%)	22 (2.2%)	0.12	-
Over 65 years of age	513 (51.8%)	59 (6.0%)	0.21	-	532 (53.7%)	40 (4.0%)	<0.01	2.44 [1.61, 3.70]
Aboriginal & Torres Strait Islander peoples	7 (0.7%)	0	1.00	-	7 (0.7%)	0	1.00	-
					313 (31.6%)			

Pregnancy	314 (31.7%)	30 (3.0%)	0.05	-	30 (3.0%)	31 (3.1%)	0.24	-
Non-English speaking	30 (3.0%)	3 (0.3%)	0.67	-	452 (45.7%)	3 (0.0%)	0.77	-
Sport injuries	448 (45.3%)	53 (5.4%)	0.40	-	100 (10.1%)	49 (4.9%)	0.39	-
Worker injury (compensable)	93 (9.4%)	10 (1.0%)	0.56	-	314 (31.7%)	3 (0.3%)	<0.01	4.36 [1.36, 14.02]
Work injury (non-compensable)	298 (30.1%)	43 (4.3%)	0.38	-	52 (5.3%)	27 (2.7%)	0.04	1.58 [1.00, 2.51]
Traffic injury (compensable)	47 (4.8%)	7 (0.7%)	0.71	-	109 (11.0%)	2 (0.2%)	0.11	-
Traffic injury (non-compensable)	105 (10.6%)	9 (0.9%)	0.29	-	76 (7.7%)	5 (0.5%)	0.02	2.75 [1.10, 6.92]
Post-surgery	72 (7.3%)	7 (0.7%)	0.47	-		3 (0.3%)	0.05	-
**Manual therapy (use 'often')**								
Counterstrain	375 (37.9%)	45 (4.5%)	0.50	-	378 (38.2%)	42 (4.2%)	0.54	-
Muscle energy technique	703 (70.9%)	85 (8.6%)	0.16	-	708 (71.4%)	80 (8.1%)	0.27	-
High-velocity, low-amplitude	555 (56.0%)	77 (7.8%)	0.37	-	569 (57.4%)	63 (6.3%)	0.32	-
manipulation					358 (36.2%)			
Joint manipulation	347 (35.1%)	46 (4.7%)	0.89	-	762 (77.0%)	35 (3.5%)	0.13	-
Soft tissue technique	748 (75.6%)	100 (10.1%)	0.36	-	554 (56.0%)	86 (8.7%)	0.24	-
Myofascial release	553 (55.9%)	59 (6.0%)	0.02	1.60 [1.08, 2.36]	83 (8.4%)	58 (5.9%)	0.11	-
Visceral techniques	87 (8.8%)	11 (1.1%)	0.93	-	77 (7.8%)	15 (1.5%)	0.12	-
Lymphatic pump	78 (7.9%)	6 (0.6%)	0.19	-	146 (14.7%)	7 (0.7%)	0.46	-
Autonomic balancing	145 (14.6%)	12 (1.2%)	0.10	-	136 (13.7%)	11 (1.1%)	0.10	-
Biodynamics	140 (14.1%)	15 (1.5%)	0.44	-	243 (24.5%)	19 (1.9%)	0.49	-
Functional technique	238 (24.0%)	32 (3.2%)	0.83	-	312 (31.5%)	27 (2.7%)	0.66	-
Balanced ligamentous tension	311 (31.4%)	38 (3.8%)	0.65	-	21 (2.1%)	37 (3.7%)	0.94	-
Chapman's reflexes	23 (2.3%)	1 (0.1%)	0.51	-	224 (22.6%)	3 (0.3%)	0.77	-
Trigger point therapy	231 (23.3%)	27 (2.7%)	0.54	-	208 (21.0%)	34 (3.4%)	0.13	-
Osteopathy in the Cranial Field	213 (21.5%)	20 (2.0%)	0.10	-	154 (15.6%)	25 (2.5%)	0.99	-
Facilitated positional release	149 (15.1%)	17 (1.7%)	0.60	-	205 (20.7%)	12 (1.2%)	0.12	-
Dry needling	206 (20.8%)	28 (2.8%)	0.80	-	658 (66.5%)	29 (2.9%)	0.34	-
Exercise prescription	652 (65.9%)	81 (8.2%)	0.44	-	17 (1.7%)	75 (7.6%)	0.41	-
Shockwave therapy	17 (2.3%)	1 (0.1%)	0.42	-	25 (2.5%)	1 (0.1%)	0.47	-
Ultrasound	24 (2.4%)	3 (0.3%)	0.94	-	16 (1.6%)	2 (0.2%)	0.58	-
TENS	15 (1.5%)	4 (0.4%)	0.18	-	2 (0.2%)	3 (0.3%)	0.47	-
Instrument manipulation	1 (0.1%)	1 (0.1%)	0.21	-	11 (1.1%)	0	0.62	-
Instrument soft tissue	10 (1.0%)	2 (0.2%)	0.58	-	111 (11.2%)	1 (0.1%)	0.79	-
Sport taping	114 (11.5%)	8 (0.8%)	0.07	-		11 (1.1%)	0.52	-
**Expanded practice scope ('definitely')**								
Prescribing rights	228 (23.0%)	29 (2.9%)	0.90	-	231 (23.3%)	26 (2.6%)	0.73	-
Referral rights to orthopaedic surgeon	629 (63.5%)	74 (7.5%)	0.13	-	627 (63.3%)	76 (7.7%)	0.85	-
Referral rights to paediatrician	480 (48.4%)	60 (6.1%)	0.67	-	480 (48.4%)	60 (6.1%)	0.64	-
Referral rights to sports medicine	712 (71.9%)	78 (7.9%)	<0.01	2.00 [1.30, 3.08]	707 (71.4%)	83 (8.4%)	0.68	-
specialist					563 (56.8%)			
Referral rights to rheumatologist	561 (56.6%)	68 (6.9%)	0.37	-	1 (0.1%)	66 (6.7%)	0.78	-
Referral rights to other medical specialist	1 (0.1%)	0	1.00	-	739 (74.6%)	0	1.00	-

Expanded diagnostic imaging rights	733 (74.0%)	89 (9.0%)	0.14	-		83 (8.4%)	0.18	-
**Research (‘strongly agree’)**								
Help patients understand osteopathy	387 (39.0%)	56 (5.6%)	0.31	-	389 (39.2%)	54 (5.4%)	0.17	-
Help general practitioners and other health professionals understand osteopathy	595 (62.5%)	75 (7.9%)	0.59	-	590 (62.0%)	80 (8.4%)	0.40	0.59 [0.36, 0.98]
Provide scientific evidence	458 (48.8%)	57 (6.1%)	0.57	-	455 (48.5%)	60 (6.4%)	0.33	-
Irrelevant to the development of osteopathy*	499 (53.3%)	64 (6.8%)	0.85	-	496 (52.9%)	67 (7.2%)	0.13	-

* ‘strongly disagree’; compensable – patient receives care paid/part-paid for by a third party payment scheme; non-compensable – patient pays for their own care with no rebate through a third-party scheme
